# Learning health systems: Driving real‐world impact in mental health and substance use disorder research

**DOI:** 10.1096/fba.2020-00124

**Published:** 2021-04-07

**Authors:** Amy M. Kilbourne, Emily Evans, David Atkins

**Affiliations:** ^1^ Health Services Research and Development Office of Research and Development Veterans Health Administration U.S. Department of Veterans Affairs Washington DC USA; ^2^ Department of Learning Health Sciences University of Michigan Ann Arbor MI USA

**Keywords:** chronic disease management, implementation science, learning health systems, mental disorders, substance use disorders, Veterans

## Abstract

The Veterans Health Administration (VHA), under the U.S. Department of Veterans Affairs (VA), is one of the largest single providers of health care in the U.S. VA supports an embedded research program that addresses VA clinical priorities in close partnership with operations leaders, which is a hallmark of a Learning Health System (LHS). Using the LHS framework, we describe current VA research initiatives in mental health and substance use disorders that rigorously evaluate national programs and policies designed to reduce the risk of suicide and opioid use disorder (data to knowledge); test implementation strategies to improve the spread of effective programs for Veterans at risk of suicide or opioid use disorder (knowledge to performance); and identify novel research directions in suicide prevention and opioid/pain treatments emanating from implementation and quality improvement research (performance to data). Lessons learned are encapsulated into best practices for building and sustaining an LHS within health systems, including the need for early engagement with clinical leaders; pragmatic research questions that focus on continuous improvement; multi‐level, ongoing input from regional and local stakeholders, and business case analyses to inform ongoing investment in sustainable infrastructure to maintain the research‐health system partnership. Essential ingredients for supporting VA as an LHS include data and information sharing capacity, protected time for researchers and leaders, and governance structures to enhance health system ownership of research findings. For researchers, incentives to work with health systems operations (e.g., retainer funding) are vital for LHS research to be recognized and valued by academic promotion committees.

## 1 BACKGROUND

There is a well‐documented disconnect between health research and practice. Research findings often take years, if not decades, to be translated into routine clinical practice.^1^ Research may not address the urgent needs of health systems, providers, or patients.^2^ A recent report from the National Academy of Medicine on the Future of Health Services Research^3^ points to the lack of alignment between academic research priorities and the needs of health systems and recommends that health researchers do more to address real‐ world problems identified by health systems, providers, patients, and other stakeholders.

This gap between research and practice, and clinical care delivery challenges that are not addressed by existing research, is especially problematic for persons suffering from noncommunicable diseases (NCDs), including chronic conditions such as mental health or substance use disorders. Mental health and substance use disorders are often considered “index” chronic conditions given the need for ongoing, coordinated care management across different providers.^4^ Mental health (e.g., major depression, schizophrenia) and substance use disorders (e.g., alcohol, tobacco, and opioid use) are also significant risk factors for other chronic illnesses, notably cardiovascular disease and lung cancer.^5^, ^6^ Nearly half of the U.S. population has experienced a mental health or substance use disorder in their lifetime,^7^ yet only a third receive adequate treatment.^8^ Lack of treatment for these conditions can lead to preventable hospitalizations or even death from suicide.^9^ Moreover, only a third of frontline providers have access to training in effective interventions for mental health or substance use disorders.^10^ Many effective interventions that are designed and tested in academic research settings may not be practical for lower‐resourced clinical settings.^11^ These barriers have been exacerbated by the COVID‐19 pandemic, which has caused delays in needed medical and mental health care and exacerbated the effects of social isolation.

Improving the quality and outcomes of care for mental health and substance use disorders requires redirecting research investments toward real‐world health care delivery problems and aligning incentives for researchers to work closely with health systems. However, the traditional metrics for advancing as an academic researcher emphasize research productivity based on grants and publications rather than health system impacts. This system does not incentivize researchers to pursue directions that may better address the needs of the health systems or populations they serve,^12^ nor does it produce sufficiently timely results for the health systems or patients in need.

The Learning Health System (LHS) framework aligns researcher and health system incentives and priorities to increase the impact of research findings and interventions on health care and outcomes. The National Academy of Medicine has defined the LHS as a system in which “science, informatics, incentives, and culture are aligned for continuous improvement and innovation, with best practices seamlessly embedded in the delivery process and new knowledge captured as an integral by‐product of the delivery experience”.^13^ A fundamental mechanism of an LHS is embedding research (and researchers) within the health system to improve the impact and timeliness of research and facilitate uptake and implementation of the evidence generated. Embedded, or partnered, research is defined as the process by which researchers and health system (operations) partners “work together, with different roles, to use research both to solve practical problems and contribute to science”.^14^, ^15^

For these research‐operations partnerships to successfully address health care priorities related to NCDs, incentives, timing, and agendas of researchers and health system leaders need to be aligned. Current research funding mechanisms do not generally provide sustained support – for both resources and protected time – to successfully build and support the partnerships, data infrastructure, and rapid evaluations of current best practices needed to ensure an efficient and responsive research enterprise that results in meaningful impacts in clinical outcomes and care delivery.^16^

This paper describes the Office of Research Development's (ORD’s) efforts in the U.S. Department of Veterans Affairs (VA) to build an LHS, particularly in the areas of mental health and substance use disorder treatment, through funding mechanisms that align researcher and operations partner priorities and infrastructure needs. VA is a Cabinet‐level agency within the Executive Branch of the U.S. Government, within which the Veterans Health Administration is responsible for delivering health care to over 9 million Veterans. In addition to clinical care, its primary missions include research and clinician education/training. Over the past decade, VA has prioritized research focused on suicide prevention and opioid use disorder treatment.^17^ As one of the largest providers of mental health and substance use disorder care in the U.S., and with the presence of an embedded research program that employs researchers affiliated with academic institutions, VA provides a unique example of building an LHS through research‐operations partnerships with support from research funding mechanisms.^18^ We discuss the lessons learned from these partnered research‐operations initiatives and how they might inform the LHS community within and external to VA, especially concerning improving outcomes in mental health and substance use disorders.

## 2 SUICIDE PREVENTION AND OPIOID USE DISORDER IN THE VA

Suicide rates for veterans are higher than for the general population, and suicide prevention is a high priority for VA. Between 2005 and 2017, 78,875 veterans died by suicide,^19^ which is more than the number of Americans killed in each major conflict except for World War II and the Civil War. Evidence suggests^20^ that the strongest predictors of death by suicide, either during service or post‐military separation, include current and past diagnoses of self‐inflicted injuries, major depression, bipolar disorder, substance use disorder, and other mental health conditions. Similarly, opioid use disorder (OUD) is a major cause of illness and death among Veterans.^21^, ^22^ Hence, ending suicide and opioid use disorder are major U.S. national initiatives.^23^, ^24^

## 3 VA LEARNING HEALTH SYSTEM FRAMEWORK

**FIGURE 1:**
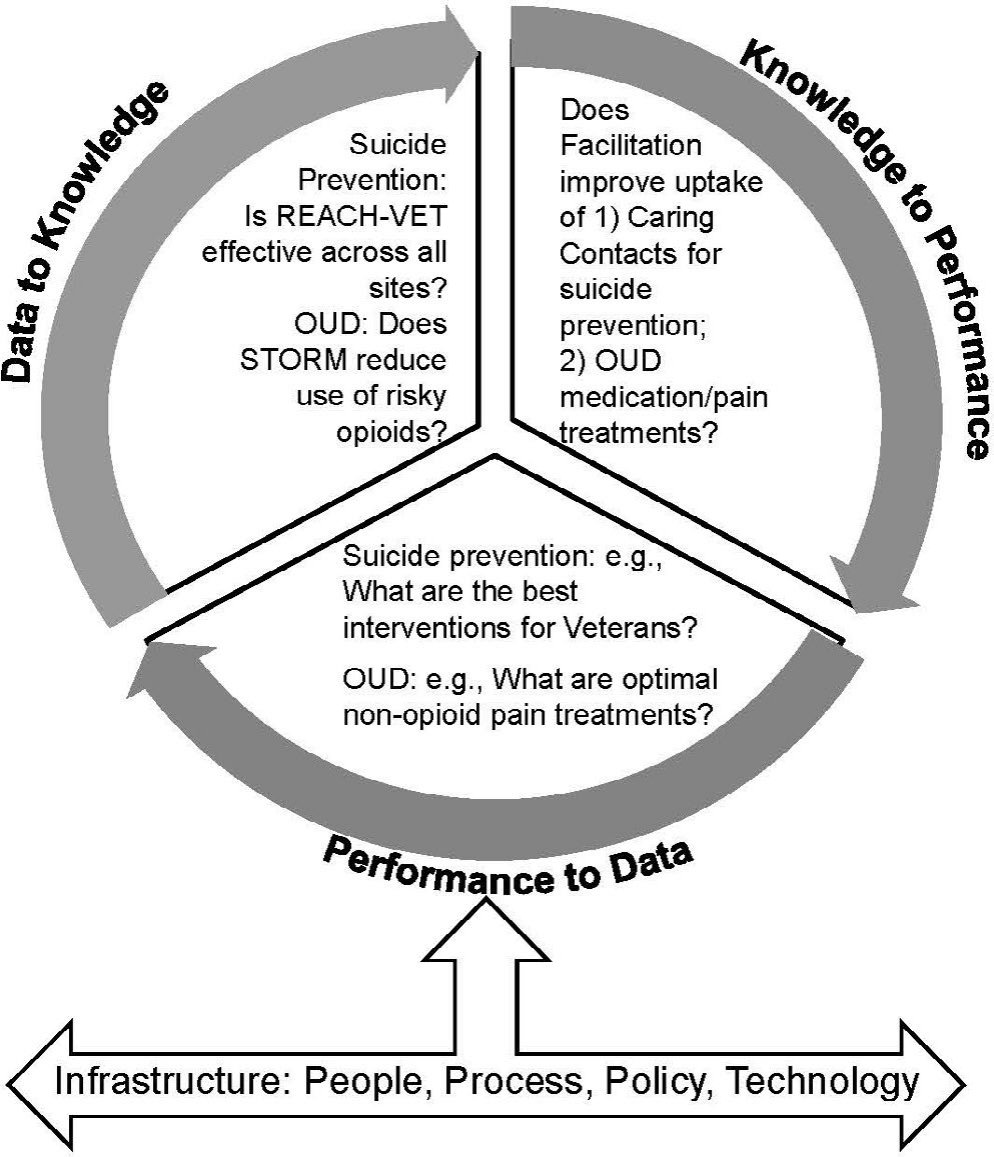
VA Learning Health System Cycle: Suicide Prevention & OUD. Adapted from Friedman et al. Legend: OUD: Opioid use disorder, STORM: Stratification Tool for Opioid Risk Management, REACH‐Vet: Recovery Engagement and Coordination for Health – Veterans Enhanced Treatment

Figure 1 provides an outline of VA’s LHS framework, adapted from previous frameworks.^25^, ^26^ It involves a progressive, three‐phase approach (data to knowledge, knowledge to performance, performance to data) that aligns people, processes, technologies, and policies to achieve continuous scientific learning in a health system:

- People include researchers, clinical operational leaders, patients, frontline providers, and other stakeholders who collaborate on a shared plan to address a clinical priority goal.
- Processes include implementation of quality improvement (Q.I.) strategies and rapid‐cycle evaluation methods to test and validate interventions in real‐world care delivery settings. Implementation strategies are methods designed to improve the quality of care by promoting uptake of evidence‐based practices among frontline providers, sites, and health systems by overcoming multilevel barriers to uptake such as costs (and other resources), care coordination, and operationalization of services.
- Technology includes electronic data captured in real‐time on clinical, patient, provider, and system‐level outcomes. In the VA, the principal source of electronic data is the Corporate Data Warehouse, which is a compilation of longitudinal electronic health record data, including inpatient, outpatient, and emergency department visits, laboratory results, medications/treatments, diagnoses, and procedures on users of the VA health care system across all VA medical centers and community‐based outpatient clinics. More limited administrative data including utilization, medications and diagnoses are available for care provided outside VA, either paid by Medicare for eligible older Veterans or paid by VA under a program to ensure more timely and convenient access to specific services.^27^
- Policies include governance for promoting continuous quality improvement, data ascertainment, curation, and analysis, and scientific discovery over time. In VA, governance is supported by the establishment of national resource centers to facilitate data infrastructure. For example, the VA Health Services Research and Development (HSR&D) program funds the VA Information Resource Center (VIREC),^28^ which supports researchers in accessing and using VA data for research and quality improvement purposes, as well as the. the Health Economics Resource Center (HERC)^28^ which supports researchers in determining the cost of VA care, assessing cost‐effectiveness, and evaluating the efficiency of VA programs and providers using common data elements.

Below we describe VA research initiatives that align with the three LHS phases for VA’s priority goals of reducing the adverse impact of NCDs, focusing on mental health, substance (opioid) use, and suicide prevention. Lessons learned from these current initiatives can help inform a process for researchers and health system leaders to continue implementing core components of the LHS for clinical priorities going forward.

## 4 DATA TO KNOWLEDGE: IDENTIFYING AND UNDERSTANDING DRIVERS OF SUICIDE AND OUD TO OPTIMIZE PREVENTION EFFORTS

Data to knowledge involves the process of identifying and understanding determinants of health and health care quality gaps and evaluating interventions that might reduce or eliminate these gaps. VA national program offices such as the Office of Mental Health and Suicide Prevention (OMHSP) have used longitudinal electronic health record data to identify and understand the drivers of suicide and opioid use disorder risk, assess adherence to evidence‐based guidelines, track variation in practice, monitor performance, and reward improvement.^29^ Data to knowledge also involves careful evaluation of interventions that are effective in real‐world practice. Strong partnerships enabled these analyses conducted by embedded researchers to understand determinants of increased risk for both suicide^30^ and opioid use disorder risk factors,^31^ which informed national interventions described below.

### 4.1 Reduce/Eliminate quality gaps: VA national program evaluations

OMHSP developed two data‐driven national interventions to identify patients at the highest risk for suicide (REACH Vet) and opioid use disorder and overdose risk (STORM) (Table 1). Recognizing the opportunity to conduct embedded research to determine the effectiveness of these programs in real‐world settings, HSR&D launched a program to evaluate national rollouts of these two interventions with additional funding from the Office of Management and Budget (OMB), which strongly encouraged randomization to produce rigorous evaluations of new government programs.^32^ Evaluation priorities focused on suicide prevention and opioid misuse were selected based on a nomination process where VA clinical leaders submitted to HSR&D priority evaluation topics, which were peer‐reviewed for their impact, clinical relevance, and feasibility of building randomization into the rollout. Researchers then submitted evaluation funding proposals to HSR&D on these topics, who scientifically peer‐reviewed them and funded the top‐scoring proposals.

**TABLE 1:** Learning health system‐focused initiatives in the VA for mental health and substance use disorders

LHS phase and initiative	Mental health priority goal: suicide prevention	Substance use disorders priority goal: opioid use disorder
Data to Knowledge National data from VA national clinical leaders identified gaps in quality Program evaluations selected by leaders and led by researchers test interventions to reduce gaps in quality/outcomes	Identify Gaps: Lack of services for high‐risk Veterans Recovery Engagement and Coordination for Health ‐ Veterans Enhanced Treatment (REACH VET): Coordinators supported Veterans in the top 0.1% risk of suicide based on a national data algorithm and coordinated care.	Identify Gaps: Lack of access to the effective management of opioid use disorder Stratification Tool for Opioid Risk Management (STORM), which uses a real‐ time data dashboard to present individual patients’ level of risk, display patient‐ specific clinical risk factors, and track the use of recommended risk mitigation strategies
Knowledge to Performance Partnered Implementation Initiatives funded through quality improvement (VA Quality Enhancement Research Initiative) designed to improve quality for clinical priorities selected by health system leaders	Caring Contacts for Suicide Prevention in emergency department settings Caring Contacts involves mailing brief, non‐demanding expressions of care and concern over a year to Veterans screened for suicide risk	Partnered Implementation Initiative: Consortium to Disseminate and Understand Implementation of Opioid Use Disorder Treatment (CONDUIT). CONDUIT used implementation strategies to scale up and spread medication‐ assisted treatment for opioid use disorder as well as non‐opioid pain treatments
Performance to Data Consortia of Research (COREs) Funded through research to create a collaborative community of researchers and leaders to support infrastructure that fosters discoveries, data needs, and dissemination products	The goal of the Suicide Prevention Research Impact NeTwork (SPRINT) is to accelerate suicide prevention research that will lead to improvements in care and ultimately, reductions in suicide among Veterans	The goal of the Pain/Opioid CORE is to foster high‐quality Veteran‐centered research to improve pain care and reduce opioid‐related harms

### 4.2 REACH‐VET suicide prevention program evaluation

In 2016, VA implemented the Recovery Engagement and Coordination for Health – Veterans Enhanced Treatment (REACH VET) program to support Veterans in the top 0.1% risk of suicide based on a national data algorithm.^33^ REACH VET supported mental health coordinators at each VA facility to identify and coordinate care for them. As REACH VET was already being rolled out nationally, the evaluation was designed to determine whether the program's effects varied across sites (and if so, why), and compare a standard versus enhanced implementation strategy to support sites not fully implementing REACH VET. The standard implementation strategy included policy memos, identified a coordinator at each of the 140 participating VA healthcare sites, web‐based training, and educational and support materials. The enhanced implementation strategy is Facilitation (Table 2), which involves ongoing, individualized advice to frontline providers on implementing and embedding REACH VET into routine clinical care by overcoming organizational barriers. Evaluation of key outcomes is still in process, including a proportion of patients identified at each facility who receive the REACH VET intervention and the proportion of providers in each facility that participate in the program.

**TABLE 2:** Example of an implementation strategy (Facilitation) and application in addressing barriers to implementation and uptake of mental and substance use disorders in VA

Facilitation component	Brief description	Key barriers addressed
Identify and engage stakeholders, including organizational leaders, local provider champions, and local opinion leaders	Facilitator helps champions (those who directly deliver the evidence‐based practice at sites) identify multi‐level stakeholders to help build rapport and motivation, align site and organization leaders’ points of influence Site local opinion leaders (e.g., influencers who are not the practice champions) support local provider champions through publicity and resource‐sharing Leadership support helps align the evidence‐based practice goal with larger goals of institution and garner additional protected times for champions	Provider: Local opinion leaders can help garner support for resources and protected time for provider champions Site/clinic: Local opinion leaders can overcome site operational inertia by identifying additional champions and opportunities where the evidence‐based practice can support other competing demands at site Organizational: Leadership endorsement helps mitigate organizational lack of prioritization, competing demands, limited incentives
Performance monitoring and goal‐setting, identify process barriers, build business case	Facilitator benchmarks sites’ ongoing progress in implementing the evidence‐based practice and patient/provider outcomes, provides feedback to provider champion to build competency and confidence in delivering evidence‐based practice Monitoring over time can identify gaps and potential improvements in organizational and practice outcomes Foster organizational change through leadership advocacy and feedback	Provider: Monitoring and feedback promotes provider self‐efficacy in delivering evidence‐based practice, helps with identifying other provider champions Site/clinic: Monitoring mitigates operational barriers by identifying and overcoming gaps in care, potential positive impacts on other site functions (e.g., patient experience, quality of care) Organizational: Use data to communicate impact of evidence‐based practice on organizational priorities (e.g., patient experience, provider productivity, quality metrics, health care costs) build ongoing support
Clarify provider roles and team processes	Facilitator guides provider champions in process mapping and defining roles of providers within the site/clinic in delivering the evidence‐based practice Providers outline process for how patients receive evidence‐based practice through and who is responsible for which task/procedure Providers with support from site and organizational opinion leaders embed evidence‐based practice components into information technology system (e.g., patient identification and outcomes monitoring)	Provider: Mitigate burnout due to duplication of efforts, unbalanced burden of tasks Site/clinic: Enables identification of process streamlining or leveraging of other services Organizational: Mitigate resource constraints by leveraging existing tools and functions
Adapt intervention and clinical processes to overcome barriers	Facilitator guides provider champions to identify feasibility issues in delivering evidence‐based practice, confirm core functions of the evidence‐based practice that cannot be changed and garner local provider input on adapting mutable components such as mode of delivery (e.g., virtual, smart phone). Use rapid‐cycle testing at sites to evaluate adaptations	Provider: opportunities to adapt helps mitigate barriers including lack of time or enthusiasm Site/clinic: mitigate resistance to change by enabling site input into adaptation and through rapid‐cycle testing demonstrate how evidence‐based practice can support site functions and other services
Transition to end‐user ownership and sustainment	Site provider champions, with guidance from Facilitator, work with site and organizational leaders to develop an action plan including roles and responsibilities for ongoing maintenance of the evidence‐based practice implementation. Form learning collaborative among champions across sites to share progress and sustainment strategies	Providers: Build self‐efficacy in practice change and implementation Site/clinic: Mitigate drift by building in automated clinical and information technology processes for maintaining evidence‐based practice Organizational: Overcome “voltage drop” that occurs post‐study or Facilitation support by building in quality measures and performance incentives to maintain evidence‐based practice, and protected time for ongoing champions to continue monitoring and learning collaborative

### 4.3 STORM opioid treatment policy evaluation

The Stratification Tool for Opioid Risk Management (STORM) focuses on reducing harmful opioid prescribing by using a real‐time data dashboard to present individual patients’ level of risk, display patient‐specific clinical risk factors, and track the use of recommended risk mitigation strategies (e.g., naloxone kits, reduction in opioid dosage) and non‐opioid pain treatments (e.g., physical therapy) for individual patients. VA national leadership rolled out the STORM policy notice requiring VA sites to complete case reviews for patients whom STORM identifies as very high‐risk of harmful opioid use (i.e., top 1% of STORM risk scores). For the randomized evaluation,^34^ researchers randomly assigned half the sites to receive notices of required additional support and oversight if they failed to meet an established percentage of case reviews. In contrast, the other half received the policy notice only. Researchers then used a stepped‐ wedge cluster randomized design to further randomize sites to conduct case reviews for an expanded pool of patients (top 5% of STORM risk scores vs. 1%) up to 15 months after the notice was released. Primary evaluation outcomes included reducing opioid prescribing and which implementation strategies supported effectiveness across sites.

### 4.4 Challenges in deploying LHS data to knowledge

Two key challenges in supporting embedded research‐practice partnerships within LHS were timing and feasibility of randomized study designs. Health systems confronted with crises may not have the luxury of waiting for the research to be completed before acting, and researchers may not have the ability to choose their preferred study design. In those cases, quasi‐experimental study designs^35^ are an alternative for estimating effects of interventions without randomization. Key examples of such designs include non‐equivalent control group designs in which investigators use electronic health record data to compare outcomes of patients receiving or not receiving a program, controlling for potential confounders that influence program receipt and outcomes of interest. Stepped‐wedge designs (which may or may not use randomization) involve systematic roll‐out of a program to sites; investigators evaluate outcomes across specific time points until all sites receive the program. Repeated measurement at each step of the roll‐out enables each site to serve as its own control while also enabling between site comparisons. An interrupted time series involves outcomes measurements from electronic health record data across multiple periods of time prior to, during, and after program deployment.

For example, even with the opportunity to embed research into their programs, VA leaders decided to move ahead with national implementation of the REACH VET suicide prevention program; the evaluation question was therefore modified to determine which implementation strategies improved REACH VET uptake (and inform understanding of program sustainment). In addition, the VA implemented REACH‐Vet before randomization could occur among sites, and investigators modified the study to evaluate different implementation strategies. Similarly, for STORM, although implementation of the policy directive was delayed at the national level, this did not impact the ability of the researchers to randomize sites. Nonetheless, these national program evaluations produced opportunities for clinical leaders to learn what would optimize uptake across local and regional VA health systems, especially by studying optimal implementation strategies.

## 5 KNOWLEDGE TO PERFORMANCE: THE VA RESEARCH QUALITY ENHANCEMENT RESEARCH INITIATIVE PARTNERED IMPLEMENTATION INITIATIVES

Knowledge to performance focuses on implementing effective interventions into real‐world care settings through a process that informs strategies to help sustain the quality improvement gains over time. To accomplish this goal, HSR&D, through its Quality Enhancement Research Initiative (QUERI), funds Partnered Implementation Initiatives (PII) that support health system–researcher teams in applying implementation science to scale up and spread EBPs addressing the priorities selected by VA regional health system leaders with the ultimate goal of informing a quality improvement playbook for health system leaders. QUERI selects PII topics based on a bottom‐up clinical topic nomination process, where regional health system leaders from the 18 regional VA integrated service networks (VISNs) nominate clinical priorities that “keep them awake at night” and that QUERI could help address via quality improvement efforts. These leaders then select their top 2–3 priorities via a live‐voting process for QUERI to support through the PII mechanism.

To be eligible for PII funding, proposals had to be co‐led by a researcher and VISN leader who chooses the priority; deploy specific evidence‐based practices addressing the clinical priority across several sites from more than one VISN; apply specific implementation strategies designed to promote the uptake of effective interventions; and benchmark impact using VA national quality performance standards. PIIs also had to conduct a business case analysis for VISNs to sustain the implementation.^36^, ^37^, ^38^ The business case analysis assessed outcomes across multiple stakeholders (e.g., provider turnover, employee satisfaction and engagement, consumer satisfaction), in addition to health care costs to inform an implementation “playbook” that describes requirements for successful implementation over time.

The first cohort of PIIs focused on suicide prevention and opioid use disorder/pain treatments: Caring Contacts for Suicide Prevention in Non‐Mental Health Settings and Consortium to Disseminate and Understand Implementation of Opioid Use Disorder Treatment (CONDUIT). Both Caring Contacts and CONDUIT applied Facilitation to implement evidence‐based practices.

Facilitation (Table 2) is a previously established bundle of implementation strategies that helps providers address organizational barriers to evidence‐based practice uptake through interactive problem‐solving and mentorship. Derived from the Promoting Action on Research Implementation in Health Services (PARiHS) framework,^39^ Facilitation has been used in the successful implementation of evidence‐based practices across NCDs.^40^, ^41^, ^42^ Core components of Facilitation^43^, ^44^ are detailed in Table 2, including include multi‐level stakeholder engagement, ongoing monitoring and feedback, operationalization of provider and team processes, adaptation to fit local needs, and transitioning to sustainment. Together these components address common barriers to implementation of evidence‐based practices at the provider, site, and organizational levels; notably limited coordination among clinicians; lack of operationalization of the evidence‐based practice; and organizational cost considerations.

### 5.1 Caring contacts for suicide prevention partnered implementation initiative

Caring Contacts is an evidence‐based suicide prevention intervention^45^ that involves mailing brief, non‐demanding expressions of care and concern to Veterans screened for suicide risk in the emergency department. Caring Contacts is currently being implemented across 28 facilities in 9 VISNs using the REACH VET implementation strategy (virtual facilitation). Key outcomes include the number of Veterans reached and suicide related behavior, and the business case analysis will also focus on the role of geographic variation in outcomes.

### 5.2 CONDUIT: OUD/Pain partnered implementation initiative

CONDUIT’s^46^ overall goal is to expand Veterans’ access to medications for OUD and evidence‐based pain treatments through the facilitation implementation strategy. Effective medications for OUD are available, but their availability and use among Veterans varies across the VA. A key barrier is the lack of provider uptake of U.S. Drug Enforcement Agency waiver forms to enable non‐pain specialist providers to prescribe OUD medications such as buprenorphine/naloxone, methadone, and naltrexone. CONDUIT involves 6 VISNs and 57 sites and spans four care settings in the OUD continuum of care: Primary Care; Specialty Care; Acute Care (inpatient and Emergency Department); and Telehealth. The implementation strategies deployed at the CONDUIT sites include virtual facilitation, with added trained internal facilitators at local sites to embed EBPs as part of routine clinical care. Key outcomes include receipt of medication‐assisted treatment for OUD and non‐opioid pain treatment. The business case analysis will inform an implementation playbook and communication strategy for Veterans and providers.

### 5.3 Challenges with LHS knowledge to performance

The focus on rapid implementation of EBPs for suicide prevention and OUD/pain treatment limited researchers’ ability to learn as much as possible from their efforts. While the regional health system leaders welcomed the additional support from researchers, investigators lacked time to pursue new research ideas that arose out of the implementation process, or compare the different types of implementation strategies (e.g., virtual versus on‐ site facilitation). Hence, PIIs could benefit from additional infrastructure support to build lasting partnerships and create comprehensive data on outcomes and mechanisms most likely to achieve long‐lasting effects on program sustainment.

## 6 PERFORMANCE TO DATA: BUILDING SUSTAINABLE STRUCTURES TO ALIGN RESEARCH AND PROGRAM PRIORITIES CONSORTIA OF RESEARCH (CORES)

Performance to data is the process by which active efforts to improve health system quality and outcomes, guided by shared goals between researchers and clinical leaders, generate new questions or discoveries that can be the topic of additional study to inform continuous quality improvement. However, shared goals between researchers and health system leaders are not sufficient to support a successful LHS partnership. Building and sustaining effective partnerships requires trust, communication and time, all of which benefit from investment in infrastructure and mechanisms to foster this partnership. To this end, HSR&D established the Consortia of Research (COREs), the first two focusing on suicide prevention and opioid use disorder.

The CORES had five aims that support an LHS: (1) create a collaborative community of researchers; (2) review and assess the existing portfolio of research; (3) establish regular communication with clinical stakeholders to identify and align program and research priorities; (4) identify and address data needs to support improvement and research; and (5) distill and communicate important research findings back to clinical stakeholders. The COREs can help the LHS cycle run more efficiently, especially in the performance to data phase of identifying and formulating new research questions. As extra incentives for partners, the COREs solicit and support rapid‐cycle projects (analyses, rapid improvement projects, pilots) to address time‐sensitive needs. Recent COREs include the Suicide Prevention Research Impact NeTwork (SPRINT) and Pain/Opioid COREs.

### 6.1 SPRINT: Suicide prevention core

SPRINT^47^ aims to accelerate suicide prevention research that will lead to improvements in care, and ultimately, reductions in suicide among Veterans. SPRINT has built a network of suicide prevention researchers and operations partners through OMHSP focused on research related to VA’s overall public health suicide prevention strategy.^48^ Through this partnership, SPRINT worked to develop a “state of the science” inventory of information about VA and non‐VA health services suicide prevention research activities and the evidence base for suicide prevention interventions. SPRINT uses this inventory to create a focused research agenda on suicide prevention and provide recommendations for multi‐site projects. SPRINT’s research agenda leverages partnerships with communities to implement tailored, local prevention plans while also focusing on evidence‐based clinical strategies for intervention.

### 6.2 Pain/Opioid core

The Pain/Opioid CORE’s goals are to foster high‐quality, Veteran‐centered research to improve pain care and reduce opioid‐related harms.^49^ The major themes of this CORE include the need for novel pain treatments, including studies of complementary and integrative health approaches, exercise/movement, psychological and behavioral interventions, and novel OUD treatments such as long‐term opioid therapy and tapering strategies. VA leadership partners include VA’s National Pain Management program, Integrative Health Coordinating Center, Specialty Care Services, and OMHSP. The Opioid/Pain CORE built a coalition of VA leaders and researchers to develop a strategic plan for the CORE that included short‐ and long‐term goals directly addressing operational partner priorities. Start‐up funds were essential to supporting these goals by incentivizing researchers to build operational partnerships, develop capacity for research studies, and conduct evidence syntheses to support a research program in this area.

## 7 DISCUSSION

Collectively, the VA Randomized Program Evaluations, Partnered Implementation Initiatives, and Consortia of Research provide a comprehensive array of research mechanisms focused on NCDs, notably in mental health and substance use disorders. Each of these initiatives align research goals with the needs of local and national health system stakeholders. Assessments of these LHS‐inspired initiatives are still in process, but together the experiences point to several insights into building an LHS to support ongoing translation of research into practice; maintaining the capacity to do so through clinical and data infrastructures that address shared clinical priority goals; testing interventions to improve Veteran health; and discovery of new treatment directions.

Despite the comprehensive overview of VA LHS components, limitations in our review include the lack of complete information on the LHS impacts, notably on cost and quality of care over time. Nonetheless, several lessons can be learned from these LHS initiatives (outlined in Table 3). First, engagement with health systems leaders must occur as early as possible, not after a research proposal is fully developed. Partners who have a voice in setting priorities and discussing alternative ways of answering a question will be more invested in the research results. Second, while program partners want to know whether their programs “work” compared to some baseline, program partners are even more interested in making their programs work better or more consistently across different settings. Third, initiatives withstood common changes to clinical priorities by ensuring that regional leaders co‐led the work, and that the initiative included input from multi‐level partners including frontline clinicians, staff, Veterans, and family members. Fourth, while business case analysis is essential for determining the value of the implementation strategies, it needs to measure outcomes beyond quality and cost to include provider experiences and patient experiences.

**TABLE 3:** LHS core values, lessons learned, and alignment of VA research partnership

LHS value	Examples of issues/challenges	Recommended steps
Participatory Leadership and Transparency	Lack of alignment of priorities among health care leaders, frontline providers, and researchers	Identify the full set of relevant stakeholders and establish channels of communications Form study team with clinical and research expertise, with engagement from local clinical leaders/providers Specify priority questions early on from the health system's perspective that can be addressed through research
Scientific integrity	Lack of planning or resources to conduct rigorous evaluation	Rigorous application of scientific methods and evaluation best practices using pragmatic designs (e.g., cluster randomization) Obtain external review of study methods
Standards for operating based on the input of multiple stakeholders	Competing demands of health care leaders and personnel	Researchers and clinical operations leaders regularly meet, plan for sustainment Cross‐functional teams garner input from multi‐level stakeholders on study execution, sustainment Implement processes to clarify roles and data access, ensure privacy, security, and confidentiality of data
Stakeholder‐ focused	Changing health system priorities	Focus on improving health care quality and outcomes for a problem affecting the health system Formulate and refine questions of interest, plan business case analysis that captures outcomes of interest across stakeholder group, the value of implementation
Inclusiveness	Lack of communication regarding expectations and timing of research	Agreements, including memoranda of understanding, data use agreements, publication and dissemination policies, and other study implementation processes that include stakeholder preferences Clinical leaders co‐lead projects, obtain recognition as key partners in success
Adaptability	Limited time in a health care setting to invest in information technology or research infrastructures	Research funding to support infrastructures that maximize rigor such as data ascertainment and analysis Rapid and iterative design and evaluation of improvement efforts
Accessibility and Value	Lack of planning or tools for providers once the research funding ends	Develop a “definition of done” and hand‐off or ownership protocol to operations partners of research results, for researchers, route to other research funding opportunities Products disseminated and made available to clinical partners including implementation playbooks

Fifth, investment in sustainable infrastructure is essential to maintain the research‐health system partnership, especially for data and information sharing capacity, protected time for researchers and leaders, and governance structures. Different funding models are possible to encourage LHS work. In the VA, we can use research funding to support more rigorous evaluations and advance science while furthering goals of clinical partners; in other systems, clinical leaders have offered funding tied to their priority questions, with the understanding that this preliminary work can be used to leverage additional research support. Formal arrangements such as memoranda of understanding, data use agreements, and guidelines for publications and other dissemination products reinforce expectations. There needs to be pragmatic data capture from multiple sources, especially from electronic health records, that can be replicated over time and embedded into workflows. Finally, to maximize sustainment, a proactive plan to clarify responsibility for the continuing clinical processes once the research ends is vital. Similarly, researchers needed to be incentivized to work with operations partners through retainer funding or through a special research funding mechanism that their academic promotion committees recognize.

Is the VA LHS generalizable beyond the VA? Key ingredients to the VA LHS included a national electronic health record, funding or protected time for researchers, and a health system with a commitment from operational leaders to participate in research or evaluation. U.S. organizations that have these include the Health Care Systems Research Network,^16^ which is a consortium of health care systems that share common electronic data elements and an infrastructure to conduct research. Increasingly, federal research funding agencies have also funded health systems and academic health centers to support LHS components. For example, the U.S. National Institutes of Health's National Cancer Institute and National Center for Advancing Translational Sciences explore the role of implementation science, building capacity in supporting and complementing LHS in precision medicine approaches to transforming patient care.^16^, ^50^

Examples of health systems implementing these LHS core elements include New York University's Langone Health system, which employs rigorous, rapid‐cycle randomized testing of existing system wide initiatives to close ineffective programs and optimize valuable programs for consumers, clinicians, and support staff.^51^ Cincinnati Children's Hospital formed pediatric learning health systems for NCDs.^52^ Recent NIH initiatives such as the HEALing Communities initiative are also applying these LHS core elements to fund multilevel community‐based organizations in several states to reduce the opioid death rate by leveraging statewide electronic health data, building research and evaluation infrastructures across sites, and involving communities and organizations in the research and implementation process.^53^

Moreover, several U.S. policies and laws encourage operationalization of LHS principles, notably through “meaningful use” electronic health records^54^ to promote data infrastructures, as well as the 21^st^ Century CURES Act^55^ which enables the use of electronic data sources to assess the public health impacts of new treatments. The Foundations for Evidence‐based Policymaking Act of 2018^56^ also requires federal agencies to use evidence and evaluation to inform budget decisions. These initiatives point to the urgent need to prepare researchers and health systems leaders to better apply LHS principles and work together to embed research initiatives that facilitate rapid discovery, adoption, and evaluation of clinical interventions and care delivery approaches.

## 8 CONCLUSIONS

Closing the gap between research and practice, especially for non‐communicable diseases such as mental health and substance use disorders, requires a concerted effort to coordinate across different stakeholders, shared goals across practitioners and researchers, data infrastructure, and commitment to make continuous learning (quality improvement and research) feasible and robust. Over the past decade, the VA has rapidly evolved to embody these core values of a Learning Health System and implement initiatives that can inform the future of clinical research and operations to ultimately improve chronic illness outcomes and care experiences for patients, especially for mental health and substance use disorders.

## CONFLICT OF INTEREST

The authors declare no conflicts of interest.

## AUTHOR CONTRIBUTIONS

A.M.K. drafted the manuscript; D.A. included current research and funding initiatives; E.E. provided critical edits and content related to the study findings. All authors reviewed and approved the manuscript's content.
